# The Impact of β-1,4-Galactosyltransferase V on Microglial Function

**DOI:** 10.3389/fncel.2021.723308

**Published:** 2021-09-03

**Authors:** Xiaoyu Wang, Naiqi Shi, Meiqi Hui, Hui Jin, Shumei Gao, Qiao Zhou, Li Zhang, Meijuan Yan, Hongmei Shen

**Affiliations:** ^1^Key Laboratory of Neuroregeneration of Jiangsu and Ministry of Education, Co-innovation Center of Neuroregeneration, Nantong University, Nantong, China; ^2^Department of Aoyang Cancer Institute, Affiliated Aoyang Hospital of Jiangsu University, Suzhou, China; ^3^School of Chemistry and Molecular Biosciences, The University of Queensland, Brisbane, QLD, Australia

**Keywords:** β-1, 4-Galactosyltransferase V, lipopolysaccharide, microglia, migration, proliferation, tumor necrosis factor receptor, interleukin-1β

## Abstract

β-1,4 Galactosyltransferase V (β-1,4-GalT V) belongs to the β-1,4 galactosyltransferase family, which modifies proteins and plays a vital role in biological function. Our previous study revealed that β-1,4-GalT V was expressed in the cortex and hippocampus and participated in the recovery of spatial learning and memory in rats with traumatic brain injury. However, the expression of β-1,4-GalT V in microglia, resident immune cells in the central nervous system, and its impact on microglia in resting and lipopolysaccharide-triggered activated stages are elusive. In this study, we clarified that β-1,4-GalT V expresses in microglia, and it regulates microglial migration, proliferation, and release of the inflammatory factors. We also observed that β-1,4-GalT V affects the expression level of tumor necrosis factor receptor (TNFR)2 instead of TNFR1. These results strongly support the fact that β-1,4-GalT V is involved in microglial function.

## Introduction

Microglial cells, the resident and primary immune cells of the central nervous system (CNS), are derived from the erythro-myeloid precursor cell that originates in the yolk sac during the early stages of development (Ginhoux et al., 2010; Casali and Reed-Geaghan, 2021). In CNS, microglia constitute approximately 5% of the total glial cell population in normal adult mice (Moehle and West, 2015), and play specialized roles in maintaining homeostasis and development in CNS, as well as in multiple neurological diseases, such as neurodegenerative diseases, neurological injury, stroke, and multiple sclerosis (Chio et al., 2015; Greenhalgh et al., 2020) due to the immune-specific functions (Jiang et al., 2021). In response to specific stimuli, such as injury, microglia rapidly migrate to the location of the injury and undergo morphological and molecular changes (Nimmerjahn et al., 2005; Aguzzi et al., 2013). The word “neuroinflammation” is specific to describe the responses of microglia in the CNS (Aguzzi et al., 2013).

Normally, microglial cells reside in a resting state which has the characteristic feature of a ramified morphology, and they play the role of immunological surveillance of the CNS (Hickey, 2001; Fan et al., 2018). Experiments in human postmortem samples show markers of microglial activity in an healthy brain, that is, in individuals lacking clinical neurological symptoms (Rogers et al., 1988; DiPatre and Gelman, 1997; Streit and Sparks, 1997), but the evidence of microglial activation was noted as a consequence of lifestyle stress (Madore et al., 2020). In neurological diseases, with diverse brain insults and a variety of neuroinflammatory stimuli, microglial cells are activated into a phagocytic state characterized by a motile ameboid form (Fan et al., 2018), and they perform the neuroinflammatory responses (Streit, 2002). Addressing the exact role of neuroinflammation in these diseases is a challenge that activated microglia to present two phenotypes, known as M1 and M2, the first being proinflammatory and the second being anti-inflammatory (Zhang et al., 2020). However, proliferation is a common accompaniment to hyperactivation (Gehrmann et al., 1995) and chronic activation of microglia (Nowacki et al., 2019), and it makes the neuroinflammatory process develop into neurotoxicity and pathogenic role for the above-mentioned neurological diseases (Kreutzberg, 1996; Nelson et al., 2002; Streit, 2004; Feng et al., 2019). The proliferation of activated microglia has been found in patients with Parkinson’s disease (PD) and animal models of PD (Neal et al., 2020). Thus, therapeutic targeting of neuroinflammation, that is microglial activation underlying neurological injury and disease pathogenesis, represents an exciting approach for novel neuroprotective strategies.

Glycosylation is an essential posttranslational modification, which determines the function of proteins and plays a critical role in important processes, such as inflammation, cellular adhesion, and endocytosis (Kouno et al., 2011; Rebelo et al., 2021). In general, glycan is biosynthesized in a stepwise manner by glycosyltransferases, and the β-1,4-galactosyltransferases (β-1,4-GalTs) family is composed of seven members (β-1,4-GalT I to VII) *in vivo* (Hennet, 2002). β-1,4-GalT V, a single-chain type II transmembrane protein, is mainly located in Golgi, and it participates in the regulation of protein glycosylated modification through the formation of the catalytic protein Gal β1 to 4 galactosyltransferase *N*-acylsphingosine (GlcNAc) chain (Shirane et al., 1999). Furthermore, β-1,4-GalT V has been cloned with cDNA from the mouse brain in the lab of Nakamura and was found to increase in postnatal expression (Nakamura et al., 2001). In our previous study, immunoblotting data confirmed that β-1,4-GalT V was expressed in the cortex and hippocampal regions in normal adult rats, and the increase of its expression improved the recovery of spatial learning and memory in rats with traumatic brain injury (TBI) (Zhang et al., 2012). Intriguingly, the expression of galactosidase β-1,4-galactosyltransferase *N*-acylsphingosine (Gal β-1,4-GlcNAc) group was found in microglia and the neurons in the cortex and hippocampus (Zhang et al., 2012). Since β-1,4-GalT V was included in the synthesis of the Gal β-1,4-GlcNAc group, we will, in the current study, verify the expression of β-1,4-GalT V in microglia and provide experimental evidence for β-1,4-GalT V as one of the dominant factors for microglial function *in vitro* and *in vivo*.

## Materials and Methods

### Constructions, Reagents, and Animals

β-1,4-galactosyltransferase V RNAi (TTCGGGACAACGTGA GGACCA) and scrambled oligonucleotide (TTCTCCGAA CGTGTCACGT) were inserted into the Age1/EcoR1 site of the hU6-MCS-Ubiquitin-EGFP-IRES-puromycin plasmid. β-1,4-GalT V cDNA and its scramble were inserted into the BamH1/Age1 site of the Ubi-MCS-3FLAG-CBh-gcGFP-IRES-puromycin plasmid. Lentivirus particles were packed using HEK-293T cells and titer determination was by using Jikai Gene Chemical Technology (Shanghai, China). The following reagents were obtained commercially: rabbit anti-β-1,4-GalT V (1:500 dilution for immunofluorescence and immunoblotting; Santa Cruz, CA, United States), rabbit anti-ionized calcium-binding adaptor molecule 1 (Iba1, 1:400 dilution for immunofluorescence; Wako, Tokyo, Japan), rabbit anti-type 1 tumor necrosis factor receptors (TNFR1, 1:400 dilution for immunofluorescence and 1:1,000 dilution for immunoblotting; Proteintech, IL, United States), rabbit anti-type 2 TNFRs (TNFR2, 1:400 dilution for immunofluorescence and 1:1,000 dilution for immunoblotting; Proteintech), mouse anti-arginase1 (Arg1, 1:1,000 dilution for immunoblotting; Proteintech), mouse anti-β-actin (1:2,000 dilution for immunoblotting; Sigma-Aldrich, Darmstadt, Germany), and Hoechst [(1:4,000 dilution for immunofluorescence and 5-ethynyl-2′-deoxyuridine (EdU)] staining (Sigma-Aldrich). Male Sprague–Dawley rats (8 weeks, 200–250 g) were obtained from the Experimental Animal Center of Nantong University (Nantong, China). Housing and breeding of animals were done in accordance with the Guidelines of China for the Care and Use of Laboratory Animals in conditions of a constant temperature and relative humidity.

### Cell Culture and Screening

Highly aggressive proliferating immortalized (HAPI) microglia cells were cultured in Roswell Park Memorial Institute (RPMI) 1640 medium (Thermo Fisher Scientific, Waltham, MA, United States) containing 10% fetal bovine serum (FBS, Thermo Fisher Scientific) and 1% antibiotic penicillin/streptomycin in a humidified 5% CO_2_/95% air incubator at 37°C. Cells were seeded in culture plates at the density of 1 × 10^5^ cells/cm^2^ in RPMI medium containing 10% FBS. After 12 h, the medium was replaced by RPMI medium and the designed EGFP-tagged lentivirus particles volume were added to cells according to the formula (lentivirus particles volume = multiplicity of infection × cell number/virus titer). At 72 h after transfection, the cells were subjected to selection at a final concentration of 1 mg/ml of puromycin in RPMI medium containing 10% FBS for 1 week. The rates of EGFP positive cells in the obtained cells were above 95% (Supplementary Figure 2), and the cells were amplified and cultured for subsequent experiments.

### ELISA Assay

After centrifugation at 12,000 RPM for 15 min, supernatants from the medium from cultured HAPI microglial cells or the cerebral cortex tissue lysate by RIPA lysis buffer (Absin Bioscience Inc., Shanghai, China) were collected, and interleukin-1β (IL-1β) and tumor necrosis factor-α (TNF-α) were assessed by ELISA sets (BD Biosciences, R&D Systems, Minneapolis, MN, United States) following the protocol of the manufacturer. The absorbance was detected at 450 nm with a 96-well microplate reader (Synergy2, Bio-TEK Instruments Inc., VT, United States). Data were normalized to the sham cultures and the lipopolysaccharide (LPS) treatment for HAPI microglia cells and cerebral cortex, respectively.

### Transwell^TM^ Migration Assay

Highly aggressive proliferating immortalized microglia cells were collected and resuspended in RPMI medium to prepare a single-cell suspension at a concentration of 1 × 10^5^ cells/ml. The cell suspension (100 μl/well) was seeded on the upper side of a Transwell^TM^ permeable supports with 8.0 μm pore polycarbonate membrane inserts (Costar, United States), which were placed in 24-well plates, and 500 μl of cell culture medium containing 10% FBS was added to the lower chamber of the culture wells. After 24 h of incubation at 37°C with 5% CO_2_, Transwell^TM^ inserts were removed carefully. The medium in the inserts was aspirated and the cells remaining on the upper surface of the inserts were gently wiped off by a cotton swab. Transwell^TM^ inserts were then fixed with 4% paraformaldehyde for 30 min at room temperature, and the cells that migrated to the lower surface were stained with 0.1% crystal violet, imaged, and counted using a DMR inverted microscope (Leica Microsystems, Bensheim, Germany). The migration index was quantified by a number of cells/the sham.

### Scratch Assay

Highly aggressive proliferating immortalized microglia cells were grown on the confluent monolayers in 6-well plates and were wounded using 200 μl pipette tips. After that, the scratched cells were washed with PBS and replaced with a 2 ml RPMI medium. For LPS treatment, the RPMI medium contained 1 μg/ml LPS. Images were taken immediately and 24 h after the cells were scratched. The migration index was quantified by the remaining gap/the initial scratched gap with ImageJ software.

### EdU Staining

Highly aggressive proliferating immortalized microglia cells were cultured in the 96-well plates, and proliferation was detected by Cell-Light EdU DNA cell proliferation kit (RiboBio, Guangzhou, China) according to the instructions of the manufacturer. Briefly, cells were incubated with a culture medium containing 50 μM EdU for 2 h, followed by fixation in 4% formaldehyde for 30 min. Then, they were decolorized and incubated with 2 mg/ml glycine for 5 min and permeabilizated with 0.5% Triton X-100 for 10 min. After extensive washing with PBS, the cells were incubated with Apollo for 30 min and then treated with Hoechst for 30 min. Images were acquired with DMR inverted microscope (Leica Microsystems). Proliferation index = (number of EdU-positive cells/number of total cells) × 100%.

### Cell Counting Kit-8 Test

Highly aggressive proliferating immortalized microglia cells were cultured in a 96-well plate, and proliferation was assessed by cell counting kit (CCK-8 kit, Dojindo Molecular Technologies, Kumamoto, Japan) according to the instructions of the manufacturer. Cells were incubated in a humidified atmosphere with 5% CO_2_ at 37°C for 2 h followed by the addition of CCK-8 solution. The absorbance was detected at 450 nm with a 96-well microplate reader as an ELISA assay. The proliferation index was quantified by normalization to the sham.

### Immunofluorescence

Highly aggressive proliferating immortalized microglia cells grown on coverslips were fixed with 4% paraformaldehyde (in PBS) for 30 min at room temperature. Following the wash, cells were incubated with primary antibodies in PBS overnight at 4°C, washed with PBS, incubated with the Cy3-conjugated anti-rabbit or mouse secondary antibodies for 2 h at room temperature. Then they were counterstained with Hoechst for 30 min, and the images were acquired with DMR inverted microscope (Leica Microsystems).

### Immunoblotting

Equal amounts of protein were detected with a bicinchoninic acid assay kit (Beyotime, Jiangsu, China) and were separated by 10% Bis-Tris-polyacrylamide electrophoresis gel. After that, the proteins were transferred to polyvinylidene difluoride (PVDF) membranes (Millipore Corp., Bedford, MA, United States), which were blocked with 5% skim milk powder and incubated with primary antibodies overnight at 4°C. The membrane was then incubated with horseradish peroxidase-linked anti-rabbit or mouse IgG (1:1,000; Santa Cruz, CA, United States) for 2 h at room temperature, and Beyo ECL Star (Beyotime, Jiangsu, China) wase used as the detection system. Data were normalized to β-actin unless it was indicated in the figure legends.

### RNA Extraction and Reverse-Transcription Polymerase Chain Reactions (RT-PCRs)

Total RNA was isolated from the cerebral cortex by RNA-quick purification kit (ES Science, Beijing, China) following the instructions of the manufacturer, and the first-strand cDNA was synthesized using a reverse transcription kit (Thermo Fisher Scientific, Waltham, Massachusetts, United States) *via* an oligo (dT_18_). We measured the expression levels of the genes by quantitative RT-PCRs (qRT-PCRs) using Light Cycler 96 and SYBR Green PCR Master Mix (Roche, Auckland, New Zealand), according to the instructions of the manufacturer. Rat gene-specific primers were obtained from Thermo Fisher Scientific (Table 1). The expression level of β-actin was used as internal control, and assays were carried out in three trials independently.

**TABLE 1:** Primers for real-time reverse-transcription polymerase chain reactions (qRT-PCRs).

Gene	Primer sequence
rat β-1,4-GalT V sense	CCCAACCACAACATTTCTTCCTG
rat β-1,4-GalT V antisense	CCAGTGTCCTCCAAGCCTGAT
rat TNF-α sense	TGCCTCAGCCTCTTCTCATTCC
rat TNF-α antisense	GGGCAGCCTTGTCCCTTGAA
rat IL-1β sense	GACTTGGGCTGTCCAGATGAGA
rat IL-1β antisense	GGAGAATACCACCACTTGTTGGCTTA
rat Arg1 sense	TGGACCCTGGGGAACACTAT
rat Arg1 antisense	GTAGCCGGGGTGAATACTGG
rat β-actin sense	CAGTTCGCCATGGATGACGATATC
rat β-actin antisense	CACGCTCGGTCAGGATCTTCATG

### Surgery

Male Sprague–Dawley rats (8 weeks, 200–250 g) were anesthetized by intraperitoneal injection of a combination of anesthetics (0.2–0.3 ml/100 g). A bilateral craniotomy was performed above the cerebral cortex. One microliter lentivirus was injected into the cerebral cortex (AP:3 mm, ML:3 mm, DV:3 mm) with a 5 μl gas-tight syringe at a speed of 200 nl/min. LPS (0.5 mg/kg) was intraperitoneally injected at the third day after intracerebral injection of lentivirus. The whole surgery protocol was approved by the Ethics Committee of Nantong University (No. 20180307-004), and all the procedures were followed according to the Guidelines of China for the Care and Use of Laboratory Animals.

### Statistical Analysis

Graphpad Prism 5 was used for statistical analysis. To evaluate differences among more than two groups, data were analyzed using one-way ANOVA for the normality and equal variance test. Student–Newman–Keuls test was used for *post hoc* analysis to identify significantly different groups, and *p* < 0.05 was considered significant. All results were produced from three independent experiments.

## Results

### Involvement of β-1,4-GalT V in Release of IL-1β Stimulated by LPS

**FIGURE 1:**
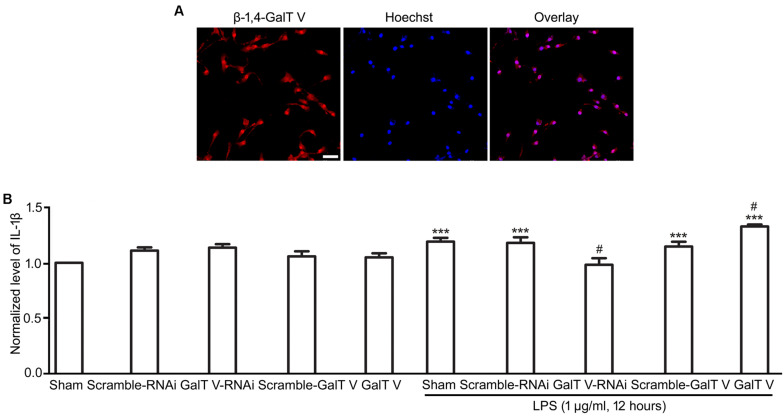
Enhancement of β-1,4-galactosyltransferase V (β-1, 4-GalT V) on the release of IL-1β evoked by LPS. β-1, 4-GalT V expressed in HAPI cells **(A)**; scale bar, 50 μm. **(B)** Quantification of IL-1β level in supernatants from HAPI cells with designated constructions as indicated were treated with LPS (1 μg/ml, 12 h). Groups significantly different from non-treated sham group, LPS-treated sham group were marked by asterisks, hashtag, respectively. Data are normalized to the non-treated Sham group, and presented as mean ± SD; *n* = 3; ***p < 0.001, ^#^*p* < 0.05.

β-1,4-galactosyltransferase V was observed in HAPI microglial cell (Figure 1A), which is a novel brain-derived microglial cell line (Cheepsunthorn et al., 2001), and expression of β-1,4-GalT V was confirmed by immunofluorescence staining in primary cultured microglia cells, which were verified by location of Iba1 (Supplementary Figure 1). To test whether β-1,4-GalT V is involved in neuroinflammation, we constructed the enhanced green fluorescent protein (EGFP) tagged β-1,4-GalT V RNAi lentivirus vector and β-1,4-GalT V overexpression lentivirus vector. The rates of EGFP positive cells in the study were above 95% and showed consistency among the designated EGFP-tag constructions (Supplementary Figures 2A,B). However, β-1,4-GalT V RNAi construction (but not a scrambled sequence) effectively knocked down endogenous β-1,4-GalT V (Supplementary Figures 2C,D), and an expression vector carrying wild type β-1, 4-GalT V gene (but not a scrambled sequence) overexpressed β-1,4-GalT V protein (Supplementary Figures 2E,F).

Lipopolysaccharide, a polysaccharide endotoxin from gram-negative bacteria, is known as a gold trigger for neuroinflammation because of its ability to bind to Toll-like receptor 4 (TLR4) in microglia (Lehnardt et al., 2003; Hanisch and Kettenmann, 2007; Catorce and Gevorkian, 2016). In the current study, we detected the release of IL-1β following treatment with 1 μg/ml LPS for 12 h. The results showed that LPS-induced secretion levels of IL-1β from HAPI microglia cells were significantly increased compared with that in the non-treated sham group (Figure 1B). β-1,4-GalT V RNAi blocked the effect of LPS on the expression level of IL-1β, but the scrambled sequence could not (Figure 1B). Furthermore, overexpression of β-1,4-GalT V intensified the release level of IL-1β notably compared with the LPS-treated sham group (Figure 1B). However, neither β-1,4-GalT V RNAi nor overexpression of β-1,4-GalT V could change the basic release of IL-1β from HAPI microglia cells (Figure 1B). These results indicated that β-1,4-GalT V is involved in the release of IL-1β, a proinflammatory molecule, from activated microglia.

### Facilitative Effect of β-1,4-GalT V on Microglia Motility

**FIGURE 2:**
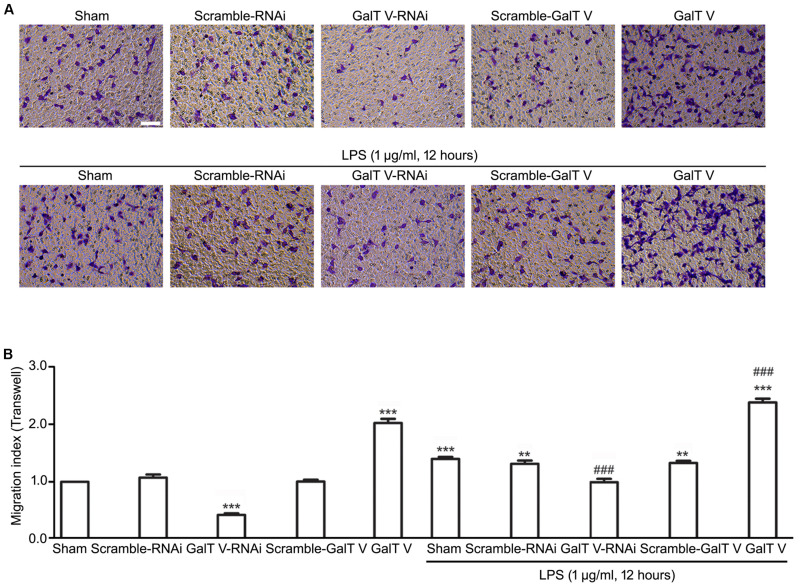
Promotion of β-1, 4-GalT V on cell migration. HAPI cells with designated constructions as indicated were treated with LPS (1 μg/ml, 12 h). **(A)** Representative images for migrated cells were labeled by crystal violet (blue); scale bar, 100 μm. **(B)** Quantification of migrated cells [migration index (Transwell) = migrated cells/migrated cells in non-treated sham group]. Groups significantly different from non-treated Sham group, LPS-treated Sham group were marked by asterisks, hashtags, respectively. Graphs show mean ± SD; *n* = 3; **p < 0.01, ***p < 0.001, ^###^*p* < 0.001.

**FIGURE 3:**
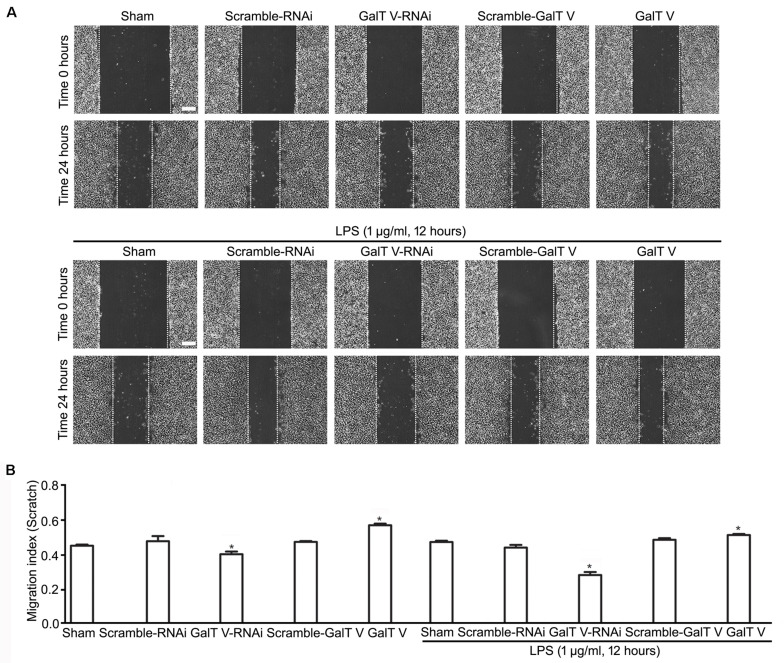
Acceleration of β-1, 4-GalT V on wound healing. HAPI cells with designated constructions as indicated were treated with LPS (1 μg/ml, 24 h). **(A)** Representative images for wound recovery; scale bar, 100 μm. **(B)** Quantification of wounded area [migration index (scratch) = wounded size after 24 h/initial wound area)]. Groups significantly different from the non-treated sham group were marked by asterisks. Graphs show mean ± SD; *n* = 3; *p < 0.05.

The increase of microglia motility by stimulation with IL-1β (Ferreira et al., 2012) prompted us to investigate the effect of β-1,4-GalT V on microglia motility. We first performed Transwell^TM^ migration assays for 24 h. Migration of β-1,4-GalT V RNAi HAPI microglial cells toward the lower compartment was significantly decreased compared with the scrambled sequence, but overexpression of β-1,4-GalT V promoted migration of HAPI microglia cells greatly (Figure 2). The data suggested that β-1,4-GalT V influences the motility of resting HAPI microglial cell. We also tested whether β-1,4-GalT V has an effect on the migration of LPS-activated HAPI microglial cells. LPS was added after 12 h to the medium in the lower chamber at the final concentration of 1 μg/ml, migration into the lower surface of Transwell^TM^ was documented. The results revealed that LPS stimulated the migration of HAPI microglial cells significantly compared with the non-treated sham, and β-1,4-GalT V RNAi restored the migration to the level of resting HAPI microglia cell (Figure 2). Furthermore, overexpression of β-1,4-GalT V promoted the migration of LPS-activated cells significantly (Figure 2). As an independent measure of HAPI microglia cells motility and to investigate the effect of β-1,4-GalT V on the migration of long-term stimulation of LPS, we performed scratch assays for 24 h for LPS treatment. Similar to Transwell^TM^ migration assays, β-1,4-GalT V caused a change of migration in the resting and the LPS-activated HAPI microglial cells (Figure 3). The data suggested that β-1,4-GalT V regulates microglial motility.

### Reduction of β-1,4-GalT V in Microglia Proliferation

**FIGURE 4:**
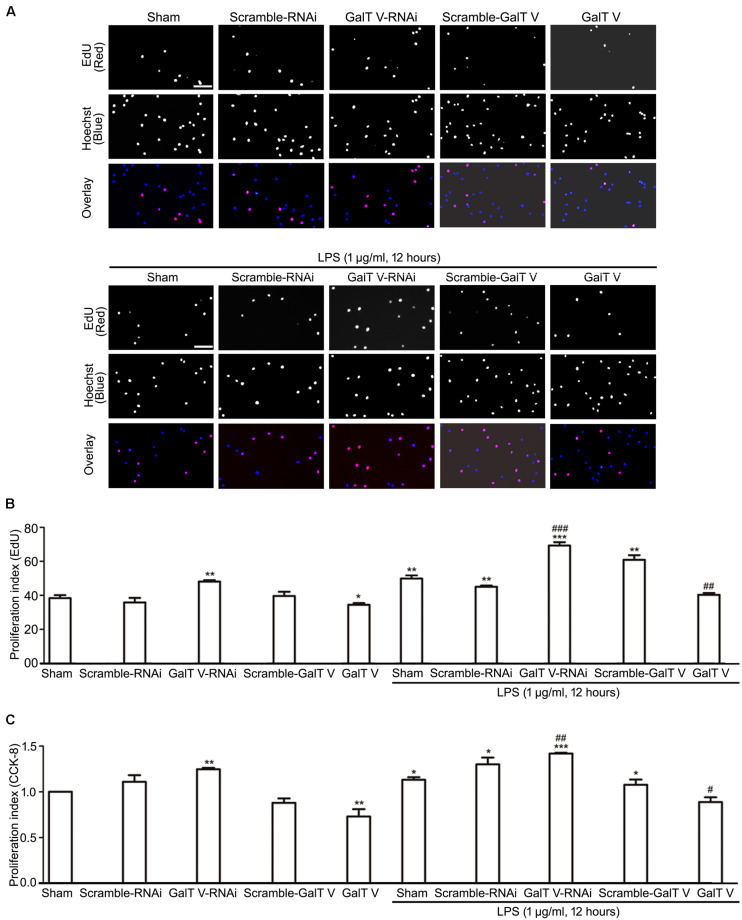
Inhibition of β-1, 4-GalT V on cell proliferation. HAPI cells with designated constructions were treated with LPS (1 μg/ml, 12 h), as indicated. Proliferation of HAPA cells was detected by EdU assay **(A,B)** and CCK-8 test **(C)**. **(A)** Representative images for DNA replicating cells by red fluorescence in nucleus; scale bar, 100 μm. **(B)** Quantification of DNA replicating cells [proliferation index (EdU) = DNA replicating cells/Hoechst positive cells × 100%]. **(C)** Quantification of cell proliferation by CCK-8 test [proliferation index (CCK-8) = absorbance/absorbance in non-treated sham group]. Groups significantly different from non-treated sham group, LPS-treated sham group were marked by asterisks, hashtags respectively. Graphs show mean ± SD; *n* = 3; *p < 0.05, **p < 0.01, ***p < 0.001, ^#^*p* < 0.05, ^##^*p* < 0.01, ^###^*p* < 0.001.

While cell proliferation and migration contribute to the increased number of resident microglia, we tested whether β-1,4-GalT V had effects on the proliferation of HAPI microglial cells. The common method to measure cell proliferation is by directly measuring new DNA synthesis, which was detected by EdU staining. β-1,4-GalT V RNAi augmented the proliferation index of HAPI microglial cells, but the scrambled sequence could not (Figures 4A,B). However, overexpression of β-1,4-GalT V lowered the proliferation index by EdU staining when opposed to the scrambled sequence (Figures 4A,B). The data indicated that proliferation can be altered by the expression of β-1,4-GalT V in resting HAPI microglial cells. Furthermore, LPS stimulation caused an increase in proliferation, which can be inhibited by the overexpression of β-1,4-GalT V and enhanced by β-1,4-GalT V knockdown (Figures 4A,B). Therefore, β-1,4-GalT V impacts the proliferation of the resting and the LPS-activated HAPI microglial cells. Similar to EdU staining, data from the CCK-8 test confirmed that β-1,4-GalT V caused a significant change of proliferation in the resting and the LPS-activated HAPI microglial cells (Figure 4C).

### Inhibition of β-1,4-GalT V on TNFR2 Expression

**FIGURE 5:**
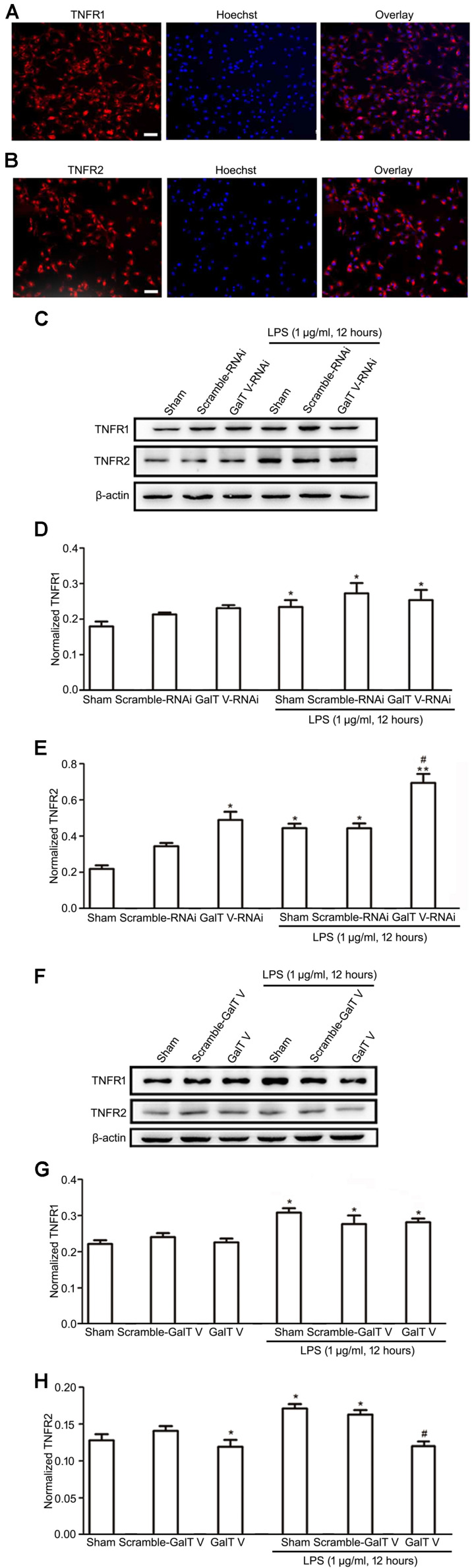
Inhibition of β-1, 4-GalT V on the expression of TNFR2 in HAPI cells. Representative images for expression of TNFR1 **(A)** and TNFR2 **(B)** by immunostaining in HAPI cells; scale bar, 50 μm. HAPI cells with constructions were treated with LPS (1 μg/ml, 12 h) as indicated **(C–H)**. **(C,F)** Representative blots of TNFR1 and TNFR2. **(D,G)** Quantification of TNFR1 for panels **(C,F),** respectively. **(E,H)** Quantification of TNFR2 for panels **(C,F),** respectively. Groups significantly different from the non-treated sham group, LPS-treated sham group were marked by asterisks, hashtag respectively. No adjustments were made for multiple comparisons. Graphs show mean ± SD; *n* = 3; *p < 0.05, **p < 0.01, ^#^*p* < 0.05.

Microglia cells express both TNFR1 and TNFR2, and TNFRs exert diverse biological functions in a context-dependent manner by distinct signaling (Dopp et al., 1997; Pan et al., 2002). However, the effect of β-1,4-GalT V on the expression of TNFRs in microglia has not been excluded. In this study, we verified that microglia express both TNFR1 and TNFR2 (Figures 5A,B) and tested whether β-1,4-GalT V altered the expression of TNFRs in the resting and the LPS-activated HAPI microglial cells. Our data showed that β-1,4-GalT V RNAi increased the expression of TNFR2, whereas overexpression of β-1,4-GalT V decreased TNFR2 expression (Figures 5C,E,F,H). In contrast, TNFR1 expression could not be modified by the overexpression of β-1,4-GalT V RNAi or β-1,4-GalT V in resting microglia cells (Figures 5C,D,F,G). To test the effect of β-1,4-GalT V on LPS-activated HAPI microglial cells, we first observed the effect of LPS on the expression of both types of TNFRs. The data showed that LPS stimulation leads to the augmentation of TNFR1 and TNFR2 expression (Figures 5C–H). However, only the TNFR2 type can be modified by β-1,4-GalT V in LPS-activated HAPI microglial cells (Figures 5C,E,F,H). That is, overexpression of β-1,4-GalT V can block the augmentation of TNFR2 expression induced by LPS, which can be enhanced by β-1,4-GalT V RNAi (Figures 5C,E,F,H). Therefore, β-1,4-GalT V can modify the expression of TNFR2 in LPS-activated microglia through LPS stimulation, which can increase in both types of TNFRs expression. Taken together, β-1,4-GalT V can suppress the expression of TNFR2 specifically in the resting and the LPS-activated HAPI microglial cells.

### Participation of β-1,4-GalT V in LPS-Induced Inflammation *in vivo*

**FIGURE 6:**
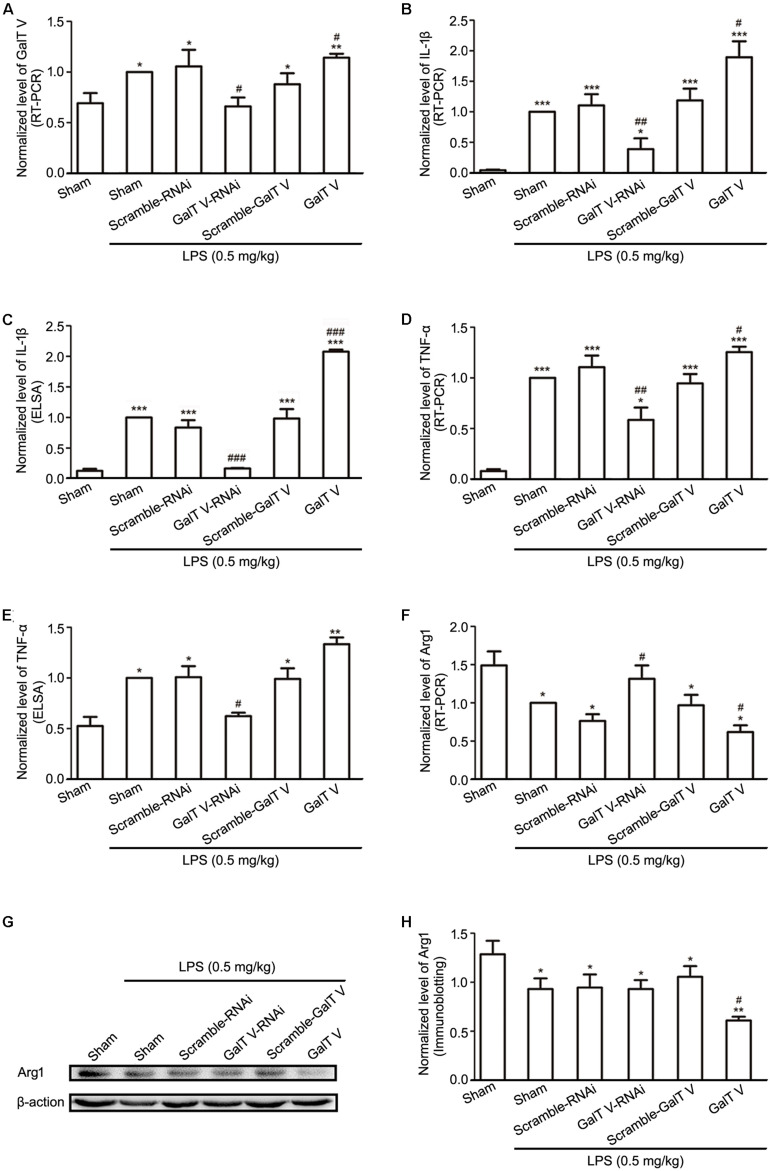
Modulation of β-1, 4-GalT V on inflammatory factors *in vivo*. SD rats wereinjected intracortical with designated lentivirus particles as indicated following intraperitoneal injection of LPS (0.5 mg/kg, 6 h) for the inflammation model. Quantification of β-1,4-GalT V **(A)**, IL-1β **(B)**, TNF-α **(D)**, Arg1 **(F)** expression in the cerebral cortex tissue by RT-PCR. **(C,E)** Quantification of IL-1β and TNF-α expression, respectively by ELSA. **(G)** Representative blots of Arg1. **(H)** Quantification of Arg1 in panel **(G)**. Groups significantly different from non-treated sham group, LPS-treated sham group were marked by asterisks, hashtags respectively. Data are expressed as mean ± SD; *n* = 3; *p < 0.05, **p < 0.01, ***p < 0.001, ^#^*p* < 0.05, ^##^*p* < 0.01, ^###^*p* < 0.001.

Since β-1,4-GalT V participated in LPS-induced inflammation *in vitro*, we examined whether β-1,4-GalT V influences the proinflammatory and anti-inflammatory factors in the rat model of systemic inflammation, which was induced by intraperitoneal injection of LPS on the third days after intracerebral injection of the designed EGFP-tagged lentivirus particles. The cerebral cortex tissues were taken from the location of intracerebral injection at 6 h after LPS intraperitoneal injection. We first observed the level of β-1,4-GalT V in the cerebral cortex by RT- PCR. The results showed that LPS systemic administration lead to an increase of β-1,4-GalT V, which was altered by injection with β-1,4-GalT V RNAi or β-1,4-GalT V overexpression lentivirus particles in the local cerebral cortex (Figure 6A). Once LPS, a neuroinflammatory stimulus, is recognized, microglia were transferred to the activated state, which is classified as classically activated microglia (M1 phenotype) or activated microglia (M2 phenotype). M1 phenotype characterized by the expression of proinflammatory molecules, such as IL-1β, TNF-α, and the expression of Arg 1, which is preferentially synthesized in microglia and does not change TNF expression, is a suitable anti-inflammatory marker for M2 phenotype (Franco and Fernandez-Suarez, 2015; Gabanyi et al., 2016; Zha et al., 2021). Therefore, the expression of IL-1β, TNF-α, and Arg 1 was tested in the same local cerebral cortex. Our data showed that the promotion of IL-β and TNF-α expression by LPS systemic administration was inhibited by β-1,4-GalT V RNAi lentivirus and strengthened by β-1,4-GalT V overexpression lentivirus (Figures 6B,C for IL-β; Figures 6D,E for TNF-α). On the contrary, LPS intraperitoneal injection resulted in the decrease of Arg1 expression, which was blocked by β-1,4-GalT V RNAi lentivirus (Figures 6F–H). Furthermore, β-1,4-GalT V overexpression lentivirus aggravated the decrease of Arg1 expression by LPS administration intraperitoneally (Figures 6F–H). Taken together, these results suggested that β-1,4-GalT V regulates the expression of proinflammatory and anti-inflammatory molecules evoked by LPS *in vivo*.

## Discussion

Although β-1,4-GalT V has been expressed in the cortex and hippocampus and implicated in neuroprotection in TBI (Nakamura et al., 2001; Zhang et al., 2012), its expression and role in microglia have remained elusive. In this study, we verified that β-1,4-GalT V was expressed in microglial cells, and it had effects on microglia migration and proliferation in the resting stage and activated stage triggered by LPS. In addition, β-1,4-GalT V knockdown restored the proinflammatory and anti-inflammatory cytokines responses of microglia by LPS *in vitro* and *in vivo*.

β-1,4-galactosyltransferases is an essential class of glycosyltransferases, which executes glycosylation of proteins by transferring the galactosidic groups from UDP-galactoside to *N*-acetylglucosamine to form galactosidase β-1,4-galactosyltransferase *N*-acylsphingosine (Gal β-1,4-GlcNAc) group (Wakselman et al., 2008). Our previous work showed that the Gal β-1,4-GlcNAc group was expressed in microglia, and β-1,4-GalT V, a member of β-1,4-GalTs, was expressed in the cortex and hippocampus (Zhang et al., 2012). However, no direct evidence proved that β-1,4-GalT V is expressed in microglia. By immunofluorescence staining, expression of β-1,4-GalT V was detected in HAPI microglial cells (Figure 1A), a novel brain-derived microglial cell line (Cheepsunthorn et al., 2001). Furthermore, β-1,4-GalT V was confirmed to be expressed in primary cultured microglia, which was identified by ionized calcium-binding adaptor molecule 1 (Iba1) (Ito et al., 1998), a characteristic protein restricted to microglia (Supplementary Figure 1). Thus, β-1,4-GalT V was expressed in microglia.

Microglia, immune-competent cells of the CNS, normally exist in a resting state and convert to an activated phenotype in response to a variety of neuroinflammatory stimuli, such as LPS, a polysaccharide endotoxin from gram-negative bacteria (Davis et al., 2017; Fan et al., 2018). To investigate the association between β-1,4-GalT V and microglial function, we constructed the screened HAPI microglial cells, in which β-1,4-GalT V was knocked down or overexpressed (Supplementary Figure 2) to test the impact of β-1,4-GalT V on microglial function in resting state and activated state, where activation was done by LPS.

Under physiological conditions, microglia are characterized by a ramified morphology, and β-1,4-GalT V did not affect the concentration of IL-1β in supernatants from HAPI microglia cells, though it modulates migration and proliferation (Figures 1B, 2–4). The results may indicate that the impact of β-1,4-GalT V on the microglial motility process is IL-1β independent in the resting state. However, microglia present a highly plastic phenotype (Davis et al., 2017). Once a neuroinflammatory stimulus is recognized, microglia are rapidly transferred to the activated state, which can be classified as classically activated microglia (M1 phenotype), or activated microglia (M2 phenotype). Activated microglia (M1) is characterized by the expression of proinflammatory molecules, such as IL-1β and TNF-α. In general, M1 microglia predominate at the site of injury and lead to progressive nerve cell damage and dysfunction (Glass et al., 2010; Ferreira et al., 2014). LPS, a polysaccharide endotoxin from gram-negative bacteria, is a potent inducer of the M1 microglia phenotype, characterized by the expression of proinflammatory molecules, such as IL-1β, TNF-α, and microglial migration and proliferation (Cohen, 2002; Rivest, 2003; Ferreira et al., 2012; Kobayashi et al., 2013). By these features, our data verified that LPS treatment triggers the M1 microglia phenotype (Figures 1–4, 6B–E) in HAPI microglial cells. Hence, LPS treatment in HAPI cells was considered as an effective protocol to test interventions for neuroinflammation.

Intriguingly, LPS evoked the increase in expression of β-1,4-GalT V, which was blocked by β-1,4-GalT V knockdown (Figure 6A). In addition, the increase of IL-1β in supernatants from HAPI microglial cells, which were induced with LPS, was eliminated by β-1,4-GalT V knockdown (Figure 1B). Similarly, β-1,4-GalT V knockdown lowered the increase of IL-1β *in vivo* (Figures 6B,C). Furthermore, β-1,4-GalT V knockdown cut down migration index, which was evoked by LPS (Figures 2, 3) owing to the increase of microglia motility by stimulation with IL-1β (Ferreira et al., 2012). However, a previous study reported the downregulation of β-1,4-GalT V, which leads to a significant increase in the proliferation of the glioma cell line SHG44 (Griciuc et al., 2013). Identical to the previous literature, we found that interfering β-1,4-GalT V promoted the proliferation of HAPI microglia cells identified by EdU and CCK-8 (Figure 4).

A pleiotropic cytokine, TNF-α, mediates many pathophysiological processes and exerts diverse biological functions in a context-dependent manner, in part, due to the presence of two distinct TNF-specific plasma membrane-localized receptors, TNFR1(CD120 a) and TNFR2(CD120b) (Wallach et al., 1999; Pan et al., 2002). Microglia, a primary source of TNF-α at CNS inflammatory sites, express both TNFR1 and TNFR2, but the signals of microglia proliferation are conducted through TNFR2 specifically (Dopp et al., 1997). We now demonstrated that HAPI microglia cells expressed both TNFR1 and TNFR2 (Figures 5A,B,C,F), and LPS augmented their expression (Figures 5C–H). However, β-1,4-GalT V performed regulation effect exclusively on TNFR2 (Figures 5E,H), though it influenced the expression of TNF-α *in vivo* (Figures 6D,E). TNFR2 canonical nuclear factor-kappa B (NF-κB) signaling may directly interpose the pathogenic proliferative response to TNF-α (Bruggeman et al., 2011). In addition, TNF-α targets the synthesis of lactosylceramide (Chatterjee et al., 2021), which is encoded by the β-1,4-GalT V gene (Yoshihara et al., 2018) and modulates the activation of microglia (Mayo et al., 2014). Thus, future analyses of TNF-α-β-1,4-GalT V-lactosylceramide signal-transduction cascade and TNF-α-NF-κB-TNFR2 axis in microglia will facilitate a complete understanding of its differential regulation effects on TNFR1 and TNFR2 in LPS-induced activated microglia. Therefore, our data support the involvement of β-1,4-GalT V in proinflammatory M1 microglia by TNF-α and IL-β thereby making an effect on LPS-induced neuroinflammation. Indeed, the anti-inflammatory M2 phenotype may prove to be an important therapeutic strategy for LPS-induced neuroinflammation. In this study, we also identified that LPS reduced the expression of Arg1, a marker for M2 microglia (Kraus et al., 2021), *in vivo*, and β-1,4-GalT V knockdown rescued the decrease of Arg1 expression induced by LPS (Figures 6F–H). Taken together, our data indicated that β-1,4-GalT V knockdown restored the secreting proinflammatory and anti-inflammatory cytokines by LPS, as well as the LPS-triggered microglia migration. In microglia proliferation, the reverse applies as a result of microglia proliferation signal *via* TNFR2 specifically. Further studies are required to determine the β-1,4-GalT V signal-transduction cascade in the LPS-mediated microglial responses.

In sum, β-1,4-GalT V exists in microglia and has an impact on microglia function, such as migration, proliferation, and release of inflammatory mediators. Combined with our previous work, β-1,4-GalT V represents a promising therapeutic strategy against TBI and other neuroinflammation-mediated diseases.

## Data Availability Statement

The raw data supporting the conclusions of this article will be made available by the authors, without undue reservation.

## Ethics Statement

The animal study was reviewed and approved by Animal Experiment Committee of Nantong University.

## Author Contributions

MY, NS, and XW designed the research. XW, NS, QZ, HJ, SG, MH, and LZ performed the research and analyzed the data. HS and XW arranged data and figures. HS, MY, NS, and XW wrote the manuscript. All the authors contributed to the article and approved the submitted version.

## Conflict of Interest

The authors declare that the research was conducted in the absence of any commercial or financial relationships that could be construed as a potential conflict of interest.

## Publisher’s Note

All claims expressed in this article are solely those of the authors and do not necessarily represent those of their affiliated organizations, or those of the publisher, the editors and the reviewers. Any product that may be evaluated in this article, or claim that may be made by its manufacturer, is not guaranteed or endorsed by the publisher.

## Supplementary Material

Supplementary Figure 1 Expression of β-1,4-galactosyltransferase V (β-1, 4-GalT V) in primary cultured microglia. Representative image for identification of microglia by Iba1 expression **(A)**; scale bar, 50 μm. Representative image for expression of β-1, 4-GalT V in primary cultured microglia cells **(B)**; scale bar, 20 μm.

Supplementary Figure 2 Efficiency of designated constructions in screened HAPI cells. Positive rates of designated EGFP-tag constructions as indicated **(A,B)**. **(A)** Representative image; scale bar, 50 μm. **(B)** Quantification of positive rates in screened cells [(EGFP positive cells/Hoechst positive cells) × 100%] in panel **(A)**; Validity of designated constructions in screened HAPI cells **(C–F)**. **(C,E)** Representative blots of β-1, 4-GalT V from screened HAPI cells as indicated. **(D,F)** Quantification of β-1, 4-GalT V for panels **(C,E),** respectively, and groups significantly different from the sham group were marked by an asterisk. No adjustments were made for multiple comparisons. The graph shows mean ± SD; *n* = 3; ^∗^*p* < 0.05, ^∗∗^*p* < 0.01.

## References

[B1] Aguzzi A. Barres B. A. Bennett M. L. (2013). Microglia: scapegoat, saboteur, or something else?. *Science* 339 156–161. 10.1126/science.1227901 23307732 PMC4431634

[B2] Bruggeman L. A. Drawz P. E. Kahoud N. Lin K. Barisoni L. Nelson P. J. (2011). TNFR2 interposes the proliferative and NF-kappaB-mediated inflammatory response by podocytes to TNF-alpha. *Lab. Invest.* 91 413–425. 10.1038/labinvest.2010.199 21221075 PMC3075956

[B3] Casali B. T. Reed-Geaghan E. G. (2021). Microglial Function and Regulation during Development, Homeostasis and Alzheimer’s Disease. *Cells* 10:957. 10.3390/cells10040957 33924200 PMC8074610

[B4] Catorce M. N. Gevorkian G. (2016). LPS-induced Murine Neuroinflammation Model: main Features and Suitability for Pre-clinical Assessment of Nutraceuticals. *Curr. Neuropharmacol.* 14 155–164. 10.2174/1570159x14666151204122017 26639457 PMC4825946

[B5] Chatterjee S. Balram A. Li W. (2021). Convergence: lactosylceramide-Centric Signaling Pathways Induce Inflammation, Oxidative Stress, and Other Phenotypic Outcomes. *Int. J. Mol. Sci.* 22:1816. 10.3390/ijms22041816 33673027 PMC7917694

[B6] Cheepsunthorn P. Radov L. Menzies S. Reid J. Connor J. R. (2001). Characterization of a novel brain-derived microglial cell line isolated from neonatal rat brain. *Glia* 35 53–62. 10.1002/glia.1070 11424192

[B7] Chio C. C. Lin M. T. Chang C. P. (2015). Microglial activation as a compelling target for treating acute traumatic brain injury. *Curr. Med. Chem.* 22 759–770. 10.2174/0929867321666141106124657 25386818

[B8] Cohen J. (2002). The immunopathogenesis of sepsis. *Nature* 420 885–891. 10.1038/nature01326 12490963

[B9] Davis B. M. Salinas-Navarro M. Cordeiro M. F. Moons L. De Groef L. (2017). Characterizing microglia activation: a spatial statistics approach to maximize information extraction. *Sci. Rep.* 7:1576. 10.1038/s41598-017-01747-8 PMC5431479 28484229

[B10] DiPatre P. L. Gelman B. B. (1997). Microglial cell activation in aging and Alzheimer disease: partial linkage with neurofibrillary tangle burden in the hippocampus. *J. Neuropathol. Exp. Neurol.* 56 143–149. 10.1097/00005072-199702000-00004 9034367

[B11] Dopp J. M. Mackenzie-Graham A. Otero G. C. Merrill J. E. (1997). Differential expression, cytokine modulation, and specific functions of type-1 and type-2 tumor necrosis factor receptors in rat glia. *J. Neuroimmunol.* 75 104–112. 10.1016/s0165-5728(97)00009-x 9143243

[B12] Fan Y. Chen Z. Pathak J. L. Carneiro A. M. D. Chung C. Y. (2018). Differential Regulation of Adhesion and Phagocytosis of Resting and Activated Microglia by Dopamine. *Front. Cell Neurosci.* 12:309. 10.3389/fncel.2018.00309 30254570 PMC6141656

[B13] Feng L. Murugan M. Bosco D. B. Liu Y. Peng J. Worrell G. A. (2019). Microglial proliferation and monocyte infiltration contribute to microgliosis following status epilepticus. *Glia* 67 1434–1448. 31179602 10.1002/glia.23616 PMC6559368

[B14] Ferreira R. Santos T. Cortes L. Cochaud S. Agasse F. Silva A. P. (2012). Neuropeptide Y inhibits interleukin-1 beta-induced microglia motility. *J. Neurochem.* 120 93–105. 10.1111/j.1471-4159.2011.07541.x 22007767

[B15] Ferreira S. T. Clarke J. R. Bomfim T. R. De Felice F. G. (2014). Inflammation, defective insulin signaling, and neuronal dysfunction in Alzheimer’s disease. *Alzheimers Dement.* 10 S76–S83. 24529528 10.1016/j.jalz.2013.12.010

[B16] Franco R. Fernandez-Suarez D. (2015). Alternatively activated microglia and macrophages in the central nervous system. *Prog. Neurobiol.* 131 65–86. 10.1016/j.pneurobio.2015.05.003 26067058

[B17] Gabanyi I. Muller P. A. Feighery L. Oliveira T. Y. Costa-Pinto F. A. Mucida D. (2016). Neuro-immune Interactions Drive Tissue Programming in Intestinal Macrophages. *Cell* 164 378–391. 10.1016/j.cell.2015.12.023 26777404 PMC4733406

[B18] Gehrmann J. Matsumoto Y. Kreutzberg G. W. (1995). Microglia: intrinsic immuneffector cell of the brain. *Brain Res. Brain Res. Rev.* 20 269–287. 10.1016/0165-0173(94)00015-h 7550361

[B19] Ginhoux F. Greter M. Leboeuf M. Nandi S. See P. Gokhan S. (2010). Fate mapping analysis reveals that adult microglia derive from primitive macrophages. *Science* 330 841–845. 10.1126/science.1194637 20966214 PMC3719181

[B20] Glass C. K. Saijo K. Winner B. Marchetto M. C. Gage F. H. (2010). Mechanisms underlying inflammation in neurodegeneration. *Cell* 140 918–934. 10.1016/j.cell.2010.02.016 20303880 PMC2873093

[B21] Greenhalgh A. D. David S. Bennett F. C. (2020). Immune cell regulation of glia during CNS injury and disease. *Nat. Rev. Neurosci.* 21 139–152. 10.1038/s41583-020-0263-9 32042145

[B22] Griciuc A. Serrano-Pozo A. Parrado A. R. Lesinski A. N. Asselin C. N. Mullin K. (2013). Alzheimer’s disease risk gene CD33 inhibits microglial uptake of amyloid beta. *Neuron* 78 631–643. 10.1016/j.neuron.2013.04.014 23623698 PMC3706457

[B23] Hanisch U. K. Kettenmann H. (2007). Microglia: active sensor and versatile effector cells in the normal and pathologic brain. *Nat. Neurosci.* 10 1387–1394. 10.1038/nn1997 17965659

[B24] Hennet T. (2002). The galactosyltransferase family. *Cell. Mol. Life Sci.* 59 1081–1095. 10.1007/s00018-002-8489-4 12222957 PMC11337546

[B25] Hickey W. F. (2001). Basic principles of immunological surveillance of the normal central nervous system. *Glia* 36 118–124. 10.1002/glia.1101 11596120

[B26] Ito D. Imai Y. Ohsawa K. Nakajima K. Fukuuchi Y. Kohsaka S. (1998). Microglia-specific localisation of a novel calcium binding protein, Iba1. *Brain Res. Mol. Brain Res.* 57 1–9. 10.1016/s0169-328x(98)00040-0 9630473

[B27] Jiang S. Wu Y. Wu S. Ye S. Kong R. Chang R. (2021). Silencing TAK1 reduces MAPKs-MMP2/9 expression to reduce inflammation-driven neurohistological disruption post spinal cord injury. *Cell Death Discov.* 7:96. 10.1038/s41420-021-00481-5 PMC8106686 33966042

[B28] Kobayashi K. Imagama S. Ohgomori T. Hirano K. Uchimura K. Sakamoto K. (2013). Minocycline selectively inhibits M1 polarization of microglia. *Cell Death Dis.* 4:e525. 10.1038/cddis.2013.54 23470532 PMC3613832

[B29] Kouno T. Kizuka Y. Nakagawa N. Yoshihara T. Asano M. Oka S. (2011). Specific enzyme complex of beta-1,4-galactosyltransferase-II and glucuronyltransferase-P facilitates biosynthesis of N-linked human natural killer-1 (HNK-1). carbohydrate. *J. Biol. Chem.* 286 31337–31346. 10.1074/jbc.m111.233353 21771787 PMC3173110

[B30] Kraus A. Buckley K. M. Salinas I. (2021). Sensing the world and its dangers: an evolutionary perspective in neuroimmunology. *Elife* 10:e66706. 10.7554/eLife.66706 PMC8075586 33900197

[B31] Kreutzberg G. W. (1996). Microglia: a sensor for pathological events in the CNS. *Trends Neurosci.* 19 312–318. 10.1016/0166-2236(96)10049-7 8843599

[B32] Lehnardt S. Massillon L. Follett P. Jensen F. E. Ratan R. Rosenberg P. A. (2003). Activation of innate immunity in the CNS triggers neurodegeneration through a Toll-like receptor 4-dependent pathway. *Proc. Natl. Acad. Sci. U. S. A.* 100 8514–8519. 10.1073/pnas.1432609100 12824464 PMC166260

[B33] Madore C. Yin Z. Leibowitz J. Butovsky O. (2020). Microglia, Lifestyle Stress, and Neurodegeneration. *Immunity* 52 222–240. 10.1016/j.immuni.2019.12.003 31924476 PMC7234821

[B34] Mayo L. Trauger S. A. Blain M. Nadeau M. Patel B. Alvarez J. I. (2014). Regulation of astrocyte activation by glycolipids drives chronic CNS inflammation. *Nat. Med.* 20 1147–1156. 10.1038/nm.3681 25216636 PMC4255949

[B35] Moehle M. S. West A. B. (2015). M1 and M2 immune activation in Parkinson’s Disease: foe and ally?. *Neuroscience* 302 59–73. 10.1016/j.neuroscience.2014.11.018 25463515 PMC4442748

[B36] Nakamura N. Yamakawa N. Sato T. Tojo H. Tachi C. Furukawa K. (2001). Differential gene expression of beta-1,4-galactosyltransferases I, II and V during mouse brain development. *J. Neurochem.* 76 29–38. 10.1046/j.1471-4159.2001.00004.x 11145975

[B37] Neal M. L. Fleming S. M. Budge K. M. Boyle A. M. Kim C. Alam G. (2020). Pharmacological inhibition of CSF1R by GW2580 reduces microglial proliferation and is protective against neuroinflammation and dopaminergic neurodegeneration. *FASEB J.* 34 1679–1694. 10.1096/fj.201900567rr 31914683 PMC7212500

[B38] Nelson P. T. Soma L. A. Lavi E. (2002). Microglia in diseases of the central nervous system. *Ann. Med.* 34 491–500. 12553488 10.1080/078538902321117698

[B39] Nimmerjahn A. Kirchhoff F. Helmchen F. (2005). Resting microglial cells are highly dynamic surveillants of brain parenchyma in vivo. *Science* 308 1314–1318. 10.1126/science.1110647 15831717

[B40] Nowacki P. Koziarska D. Masztalewicz M. (2019). Microglia and astroglia proliferation within the normal appearing white matter in histologically active and inactive multiple sclerosis. *Folia Neuropathol.* 57 249–257. 10.5114/fn.2019.88453 31588711

[B41] Pan S. An P. Zhang R. He X. Yin G. Min W. (2002). Etk/Bmx as a tumor necrosis factor receptor type 2-specific kinase: role in endothelial cell migration and angiogenesis. *Mol. Cell Biol.* 22 7512–7523. 10.1128/mcb.22.21.7512-7523.2002 12370298 PMC135657

[B42] Rebelo A. L. Gubinelli F. Roost P. Jan C. Brouillet E. Van Camp N. (2021). Complete spatial characterisation of N-glycosylation upon striatal neuroinflammation in the rodent brain. *J. Neuroinflammation* 18:116. 10.1186/s12974-021-02163-6 PMC8127229 33993882

[B43] Rivest S. (2003). Molecular insights on the cerebral innate immune system. *Brain Behav. Immun.* 17 13–19. 10.1016/s0889-1591(02)00055-7 12615045

[B44] Rogers J. Luber-Narod J. Styren S. D. Civin W. H. (1988). Expression of immune system-associated antigens by cells of the human central nervous system: relationship to the pathology of Alzheimer’s disease. *Neurobiol. Aging* 9 339–349. 10.1016/s0197-4580(88)80079-4 3263583

[B45] Shirane K. Sato T. Segawa K. Furukawa K. (1999). Involvement of beta-1,4-galactosyltransferase V in malignant transformation-associated changes in glycosylation. *Biochem. Biophys. Res. Commun.* 265 434–438. 10.1006/bbrc.1999.1684 10558885

[B46] Streit W. J. (2002). Microglia as neuroprotective, immunocompetent cells of the CNS. *Glia* 40 133–139. 10.1002/glia.10154 12379901

[B47] Streit W. J. (2004). Microglia and Alzheimer’s disease pathogenesis. *J. Neurosci. Res.* 77 1–8. 15197750 10.1002/jnr.20093

[B48] Streit W. J. Sparks D. L. (1997). Activation of microglia in the brains of humans with heart disease and hypercholesterolemic rabbits. *J. Mol. Med.* 75 130–138. 10.1007/s001090050097 9083930

[B49] Wakselman S. Béchade C. Roumier A. Bernard D. Triller A. Bessis A. (2008). Developmental neuronal death in hippocampus requires the microglial CD11b integrin and DAP12 immunoreceptor. *J. Neurosci.* 28 8138–8143. 10.1523/jneurosci.1006-08.2008 18685038 PMC6670768

[B50] Wallach D. Varfolomeev E. E. Malinin N. L. Goltsev Y. V. Kovalenko A. V. Boldin M. P. (1999). Tumor necrosis factor receptor and Fas signaling mechanisms. *Annu. Rev. Immunol.* 17 331–367. 10358762 10.1146/annurev.immunol.17.1.331

[B51] Yoshihara T. Satake H. Nishie T. Okino N. Hatta T. Otani H. (2018). Lactosylceramide synthases encoded by B4galt5 and 6 genes are pivotal for neuronal generation and myelin formation in mice. *PLoS Genet.* 14:e1007545. 10.1371/journal.pgen.1007545 30114188 PMC6095488

[B52] Zha Z. Gao Y. F. Ji J. Sun Y. Q. Li J. L. Qi F. (2021). Bu Shen Yi Sui Capsule Alleviates Neuroinflammation and Demyelination by Promoting Microglia toward M2 Polarization, Which Correlates with Changes in miR-124 and miR-155 in Experimental Autoimmune Encephalomyelitis. *Oxid. Med. Cell. Longev.* 2021:5521503. 10.1155/2021/5521503 PMC7987454 33815654

[B53] Zhang L. Liu J. Cheng C. Yuan Y. Yu B. Shen A. (2012). The neuroprotective effect of pyrroloquinoline quinone on traumatic brain injury. *J. Neurotrauma* 29 851–864. 10.1089/neu.2011.1882 22040225 PMC3303106

[B54] Zhang Z. Tian Y. Ye K. (2020). delta-secretase in neurodegenerative diseases: mechanisms, regulators and therapeutic opportunities. *Transl. Neurodegener.* 9:1. 10.1186/s40035-019-0179-3 PMC6943888 31911834

