# Supplementary figures and images for: The Impact of β-1,4-Galactosyltransferase V on Microglial Function
Source: Front Cell Neurosci. 2021 Sep 3;15:723308. doi: 10.3389/fncel.2021.723308 (PMC8446519; doi:10.3389/fncel.2021.723308)

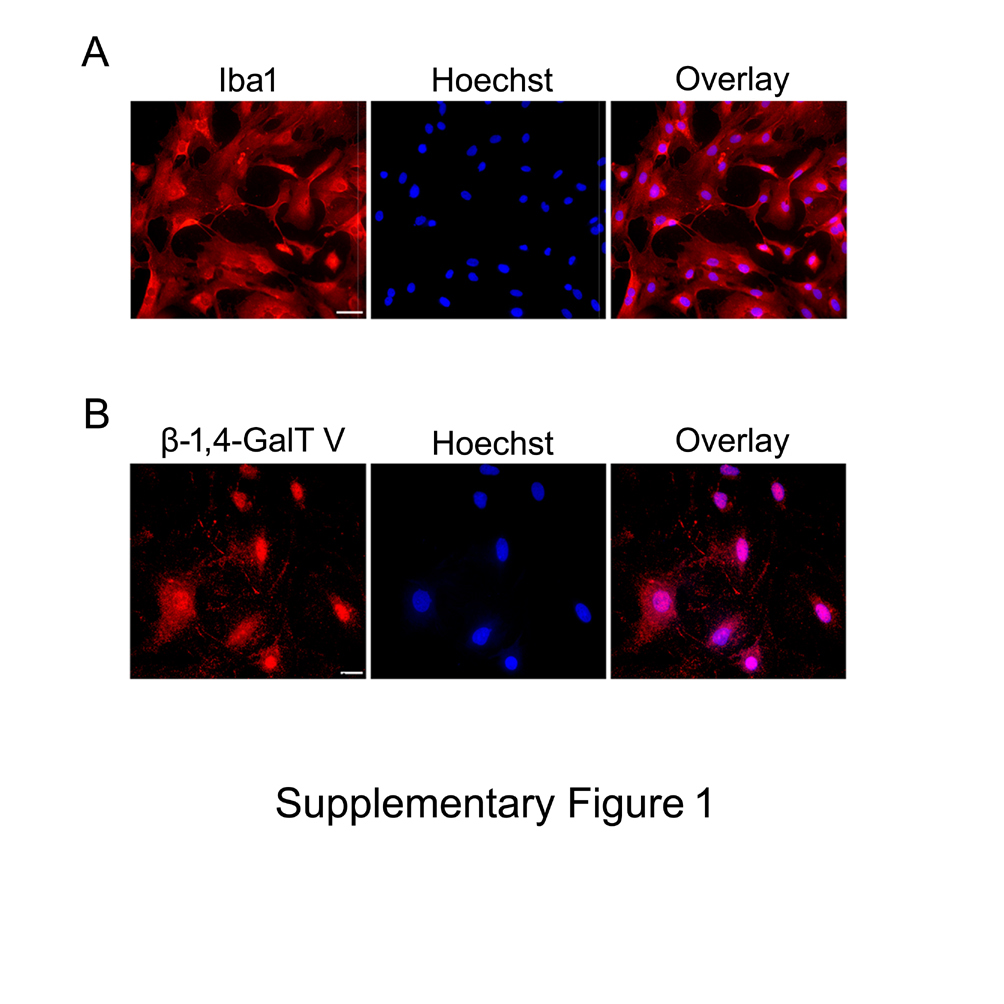

Supplement: Supplementary Figure 1 — Expression of β-1,4-galactosyltransferase V (β-1, 4-GalT V) in primary cultured microglia. Representative image for identification of microglia by Iba1 expression (A); scale bar, 50 μm. Representative image for expression of β-1, 4-GalT V in primary cultured microglia cells (B); scale bar, 20 μm. [file Image_1.jpeg]

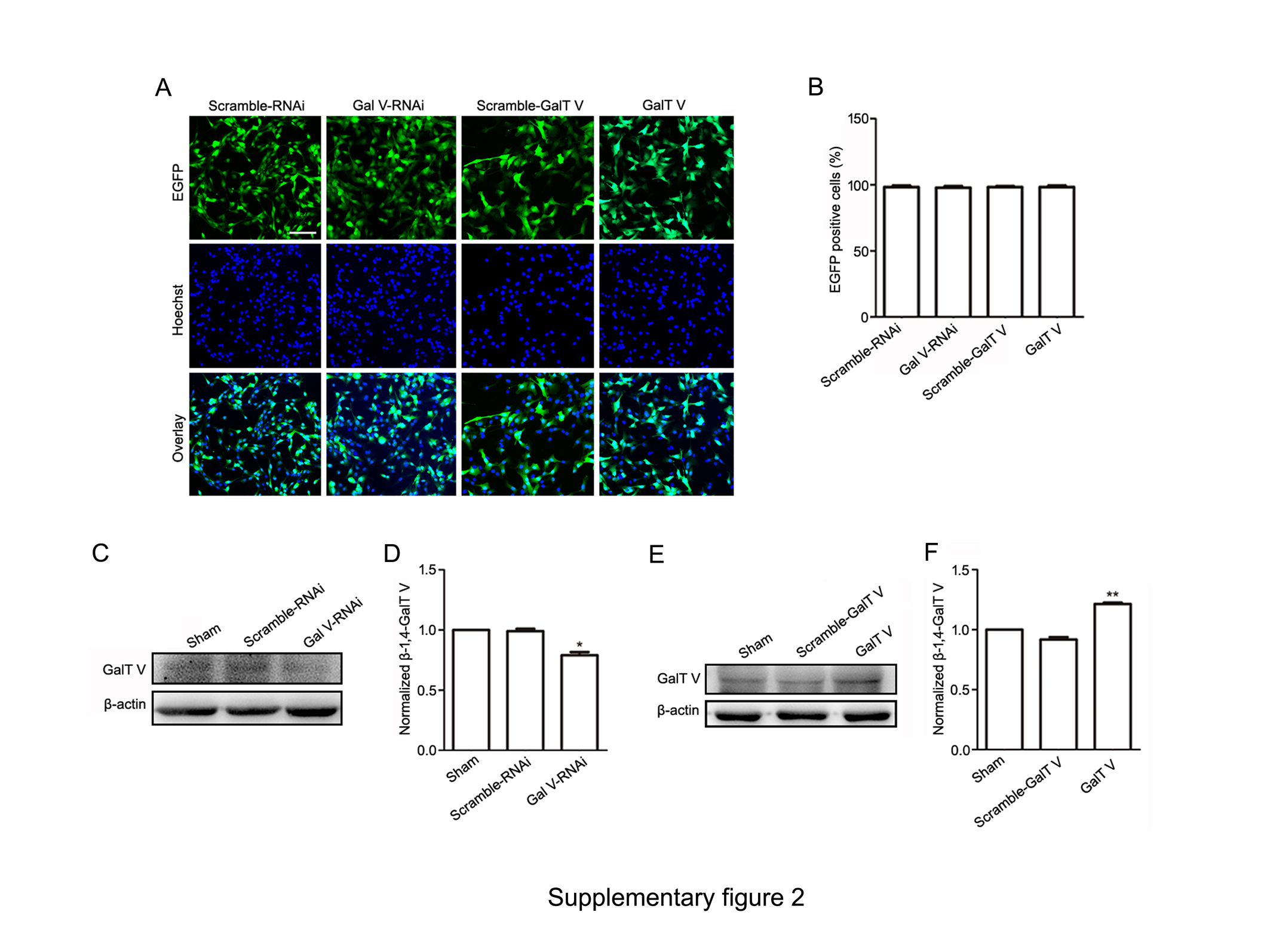

Supplement: Supplementary Figure 2 — Efficiency of designated constructions in screened HAPI cells. Positive rates of designated EGFP-tag constructions as indicated (A,B). (A) Representative image; scale bar, 50 μm. (B) Quantification of positive rates in screened cells [(EGFP positive cells/Hoechst positive cells) × 100%] in panel (A); Validity of designated constructions in screened HAPI cells (C–F). (C,E) Representative blots of β-1, 4-GalT V from screened HAPI cells as indicated. (D,F) Quantification of β-1, 4-GalT V for panels (C,E), respectively, and groups significantly different from the sham group were marked by an asterisk. No adjustments were made for multiple comparisons. The graph shows mean ± SD; n = 3; ∗p < 0.05, ∗∗p < 0.01. [file Image_2.jpeg]
